# Mathematical Models of Blast-Induced TBI: Current Status, Challenges, and Prospects

**DOI:** 10.3389/fneur.2013.00059

**Published:** 2013-05-30

**Authors:** Raj K. Gupta, Andrzej Przekwas

**Affiliations:** ^1^Department of Defense Blast Injury Research Program Coordinating Office, U.S. Army Medical Research and Materiel Command, Fort Detrick, MD, USA; ^2^Computational Medicine and Biology Division, CFD Research Corporation, Huntsville, AL, USA

**Keywords:** traumatic brain injury, blast injury, mathematical model, biomechanics, neurobiology

## Abstract

Blast-induced traumatic brain injury (TBI) has become a signature wound of recent military activities and is the leading cause of death and long-term disability among U.S. soldiers. The current limited understanding of brain injury mechanisms impedes the development of protection, diagnostic, and treatment strategies. We believe mathematical models of blast wave brain injury biomechanics and neurobiology, complemented with *in vitro* and *in vivo* experimental studies, will enable a better understanding of injury mechanisms and accelerate the development of both protective and treatment strategies. The goal of this paper is to review the current state of the art in mathematical and computational modeling of blast-induced TBI, identify research gaps, and recommend future developments. A brief overview of blast wave physics, injury biomechanics, and the neurobiology of brain injury is used as a foundation for a more detailed discussion of multiscale mathematical models of primary biomechanics and secondary injury and repair mechanisms. The paper also presents a discussion of model development strategies, experimental approaches to generate benchmark data for model validation, and potential applications of the model for prevention and protection against blast wave TBI.

## Introduction

In the current conflicts in Iraq and Afghanistan improvised explosive devices (IEDs) are frequently used weapons of adversary combatants and terrorists against U.S. troops and civilians (Ramasamy et al., 2009; Duckworth et al., 2012; Kang et al., 2012). The blast-induced traumatic brain injury (bTBI) has become a “signature wound of the war on terror” (Bhattacharjee, 2008). Consequently its mitigation, diagnosis, and management are of particular interest to the military. A recent RAND report estimates that 320,000 service members, or 20% of the deployed force, potentially suffer from TBI (Tanielian and Jaycox, 2008). Blast events account for nearly 70% of injuries in wounded service members in both Iraq and Afghanistan, and are the main cause of TBI (Okie, 2005; Heltemes et al., 2012). While penetrating and severe head injuries comprise only 2.8% of injuries, 155,623, or about 80%, were classified as mild TBI (mTBI) (Curley et al., 2011; DePalma et al., 2011). Most mTBI cases result in cognitive deficits immediately after the brain injury and only ~5% report brief loss of consciousness (Hoge et al., 2008; Ling et al., 2009). Although most of mTBI cases are expected to recover, persistent symptoms after injury, such as chronic dizziness, fatigue, headaches, and delayed recall of memory are common (Elder and Cristian, 2009; Warden et al., 2009; Heltemes et al., 2012).

In spite of immense clinical and preclinical research on impact-related brain injury due to vehicle crash and sport injuries in civilian population, current understanding of injury mechanisms is limited, diagnostics and treatment remain controversial, and little is known about the short- and long-term outcomes of mTBI. Compared to impact-related brain injury, the mechanisms involved in blast-induced mTBI are much less understood. Over the last few decades the Department of Defense (DoD) has performed substantial research on blast trauma to the body, primarily to address injuries seen in previous conflicts, and to improve personal protective equipment (PPE) (Elsayed and Atkins, 2008). The resulting improvements in the PPE and trauma care have mitigated or reduced potential blast and ballistic injury to the thorax but vulnerability to face, ear, brain, groin, and extremity injury still remain (Curley et al., 2011). Protection against blast wave TBI is particularly challenging because, in spite of the protective helmet, a significant part of the soldier’s head is still exposed to the blast. Until recently, it was not clear how a blast wave penetrates the cranium and causes brain injury and, if and how military helmets protect against it (Carey et al., 2006; Nyein et al., 2010; Przekwas et al., 2011; Zhang et al., 2011). Military helmets are traditionally designed to protect against ballistic and impact injury and even recent redesign of the sling suspension replaced by foam pads did not completely resolve blast TBI issues.

Given these uncertainties and the continued need to protect U.S. military, the DoD has made substantial research investments in understanding military relevant acute TBI and chronic mTBI (Leggieri, 2009). Several scientific teams are conducting laboratory and clinical studies to elucidate injury mechanisms and to develop protective strategies. The majority of these efforts use an experimental approach of animal testing, *in vitro* study, and analysis of clinical data, all of which are useful and necessary but are slow, expensive, lack injury scaling, and prediction capability. Better understanding of the blast wave injury mechanisms may be possible with a complementary experimental and computational modeling approach. Validated biomechanics and physiology based mathematical modeling tools of blast head injury may reduce the need for trial-and-error tests involving laboratory animals, yet provide a capability to study brain injury mechanisms, perhaps accelerating the development of neuroprotective strategies and aiding in the development of improved protective armor (Leggieri, 2009; Gupta and Przekwas, 2011).

Mathematical models of brain injury biomechanics have been developed for decades, primarily to study accidental impacts and vehicle crashes (King et al., 1995; Zhang et al., 2001; Takhounts et al., 2003; Brands et al., 2004; Kleiven, 2007). Models of explosive blast TBI are not well established yet because the injury mechanisms are not well understood and the computational methods needed to simulate these fast and multiphysics events are inadequate. The goal of this paper is to review the current state of the art in mathematical modeling of blast wave TBI, identify areas for further developments, model validation strategies, and potential applications in diagnostics, injury prevention, and protection.

## Blast Waves and Brain Injury Mechanisms

### Blast waves and blast – human body interaction

The first phase of an explosion is the *detonation*, a rapid chemical reaction and energy release generating high pressures and temperatures. The expansion of gases after detonation compresses the surrounding air into a pressure wave, *shock wave*, that propagates at supersonic speed radially in all directions from the explosion site. The front of the shock wave is followed by a high speed *blast wind*. The shock wave generated by an explosion blast is called the *blast wave*. If the explosion of a charge occurs at the ground surface the energy is released into a half hemisphere, so it generates almost two times larger effects as the free air explosion.

**Figure 1:**
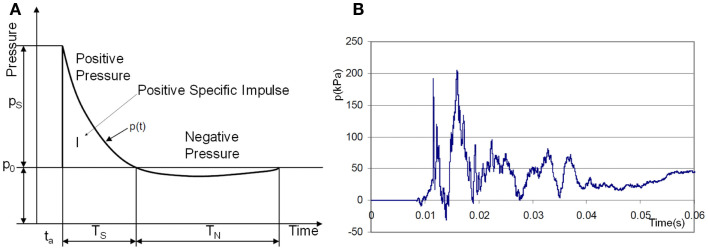
**(A)** Ideal shock wave pressure profile (Friedlander curve) and **(B)** example of complex overpressure pattern inside a vehicle subjected to a blast mine (NATO, 2007).

Theoretical and computational models often start with an idealized explosion assuming the instantaneous release of energy from a point source in a free field. In such a case an analytical solution of the Sedov–Taylor problem (Taylor, 1950) provides the pressure-time history in the form of the Friedlander curve, Figure 1A. In practice, realistic impulses differ from the ideal Friedlander profile. The effects of reflecting surfaces such as ground or walls of solid objects produce secondary reflected waves and complex wave patterns. For example, an experimental pressure trace of a blast wave inside of a military vehicle, Figure 1B (NATO, 2007), typically exhibits complex patterns with several wave reflections.

When a blast wave encounters an object of higher density, such as the human body, it reflects off of the object, diffracts around it, and passes through it in the form of elastic and shear waves (Cullis, 2002), Figure 2. The reflected wave overpressure significantly exceeds the overpressure of the incident wave. The side walls, parallel to the shock propagation direction, are loaded with the free shock overpressure. The rear side loading begins after the blast wave passes the body and the diffracted waves meet at the center back side. A person next to a solid wall may be exposed to not only the forward shock wave but also to even stronger reflected waves. In addition to the pressure loading, the body will also experience friction drag forces induced by the blast wind. This drag force appears after the primary wave front but its duration is much longer. Furthermore, blast injuries in a confined space may be more severe as the person is exposed to multiple reflected waves coming from various directions.

**Figure 2:**
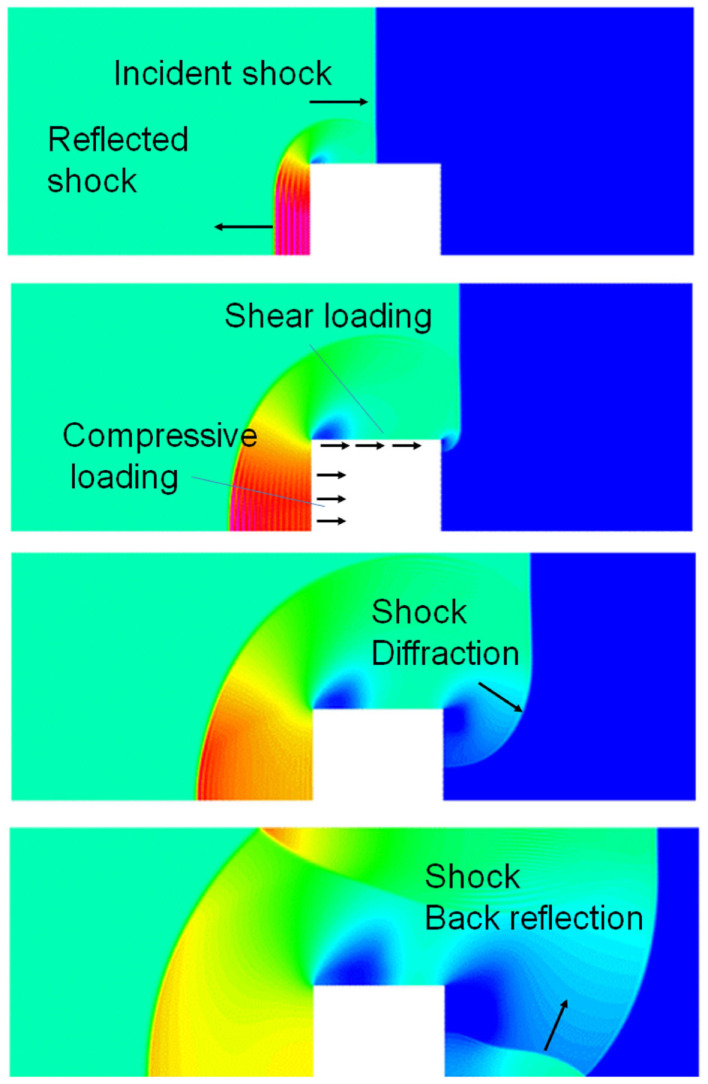
**CFD simulation of a blast wave impacting a sold block; four time instances of pressure fields showing shock reflection from the front face, diffraction around the block, and back reflection behind the block**.

From the human injury view point, the most important part of the wave energy is the one that is transmitted into the body in the form of both positive (compression) and negative (tension) stress waves as well as shear stress waves. In tissues, the steep gradient pressure waves are absorbed by viscoelastic damping and tissue plastic deformation (tearing, breaking), resulting in mechanical injury. When the pressure wave crosses material interfaces with different densities, large perturbations in stress and deformation take place. A wave impacting a denser material compresses it and when it emerges from denser to lighter material it creates tensile deformations. Therefore in the human body, organs and tissues of different densities are accelerated at different relative rates, resulting in displacement, stretching, and shearing forces (Nakagawa et al., 2011).

Based on the above physical description of the blast wave events, for the purpose of this article, we classify four potential *insults* caused by blast explosions: *primary blast insult (PBI)* due to the shock wave, *secondary blast insult* due to blast-propelled debris fragments causing blunt or penetrating ballistic trauma, and *tertiary blast insults* due to human body translocation by blast loads and the resulting impact on rigid objects. *Quaternary blast insults* refer to all other types of injury including burns, environmental wound contamination, etc. Detailed discussion of the injury mechanisms and pathophysiology of trauma for these insult types, particularly lung injury, have been described in military medicine publications and reports (Stuhmiller et al., 1999; DePalma et al., 2005, 2011; Elsayed and Atkins, 2008; Stuhmiller, 2008; Champion et al., 2009).

In the published blast injury literature the expression “primary and secondary injury” is interchangeably used to describe both insults to the body as well as tissue injury and repair mechanisms within the body. For the sake of clarity in this article we define the *insult* to describe external loads to the body and the *injury* to describe the physical, physiological, and biological mechanisms of damage and repair.

### Primary mechanisms of blast TBI

**Figure 3:**
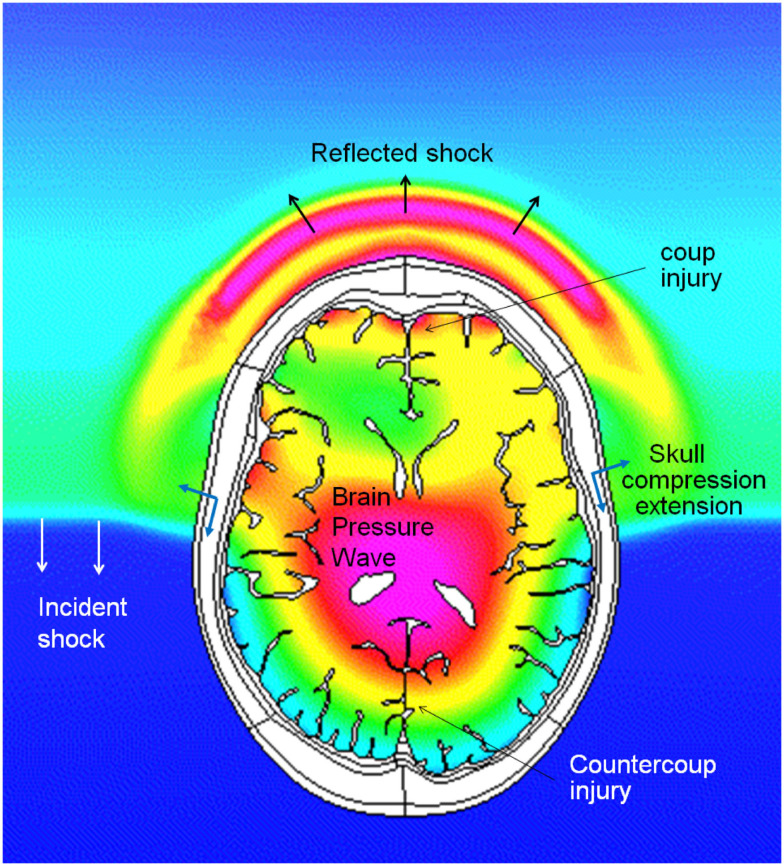
**Coupled simulations of CFD blast wave and FEM biomechanics of a human head**. Pressure profiles in the air and in the brain during intracranial pressure wave penetration. Note that the intracranial pressure wave is faster than the incident shock wave in the air.

There are several potential pathways for the blast wave energy to enter the brain, including: (1) the skull deformation creating a stress wave within the brain, (2) translation/rotation of the head causing compression/shear waves within the brain as well as brain rotation within the skull, (3) the pressure wave directly entering the brain via various foramina (orbital, ethmoidal, vestibulo-cochlear, foramen magnum, and vascular foramina), and (4) an elastic wave propagating along blood vessels from a compressed thorax (Bhattacharjee, 2008; Courtney and Courtney, 2009; Cernak, 2010; Chavko et al., 2011; Bass et al., 2012). Computational and experimental studies show that the cranial bone is a good transmitter of elastic waves with little attenuation below 10^4^–10^5^ Hz (Stenfelt and Goode, 2005). Cranial deformations are transmitted through the cerebrospinal fluid (CSF) to the brain. In the initial period of skull deformation the compression waves move through the skull and brain tissue faster than the wave in the free air, which is shown in Figure 3, presenting predicted pressure profiles in the air and in the brain during a simulated blast load (Przekwas et al., 2011). As in any elastic structure, the impulsively compressed skull, after little delay, will recoil creating a tension wave in the CSF and in the brain. This event may coincide with the arrival of the under-pressure part of the blast wave that may further exacerbate the skull/brain recompression event.

In addition to the compression/tension waves, which propagate in the skull with the speed of sound (~1560 m/s), the geometric/material asymmetries of the skull/brain and non-uniform blast loading will also generate shear waves in the brain. These shear waves are orders of magnitude slower (~10 m/s), persist longer and can be more damaging then compression waves. *In vitro* and *in vivo* experiments show that tension strains are much more damaging to the tissue than compression strains (Xi and Zhong, 2001). Intuitively it can be explained that the surrounding water resists the compression and supports tissue structural components while the tensile force directly disrupts weaker (hydrogen, van der Waals) and stronger (covalent, ionic) bonds at the molecular level.

The human brain has several deep sulci filled with CSF separating various gyri, probably to facilitate larger access area for the perfusing blood vessels. We believe that they may also have an evolutionary biomechanical protective role. As it was mentioned above, the shear waves in brain can be more injurious than compression waves. A fluid filled sulcus between two gyri is a perfect transmitter of a compression wave but it is a very poor transmitter of shear waves. Therefore, sulci may play a protective role in the brain gray matter located close to the surface of gyri during the shear (tangential) motion.

Large, rapidly changing tensions in the brain and in CSF may also cause short living cavitation spots. A collapsing cavitation gas bubble can cause extensive damage to the surrounding tissue. However, the possibility of blast wave induced cavitation in the brain remains controversial. Cavitation has been experimentally observed in laboratory experiments with idealized human head surrogates (cylindrical fluid/gel containers) loaded with high speed impactors (Kurosawa et al., 2009) or exposed to shock tube blasts (Goeller et al., 2012). Recent computational simulations of blast wave head/brain biomechanics predicted unphysical negative absolute pressures in the brain suggesting the presence of cavitation (Moore et al., 2009; Przekwas et al., 2009; Nyein et al., 2010). Others have used “cavitation models” in the form of pressure traps (Ziejewski et al., 2007; Moss et al., 2009; Panzer et al., 2012a). Negative absolute pressures are physically possible in de-gassed or entrapped liquids, e.g., in very tall trees or inside rock formations (Zheng et al., 1991), however they are less likely in the metabolically active brain tissue with relatively large concentrations of O_2_ and CO_2_ which would act as nuclei spots for cavitation.

**Figure 4:**
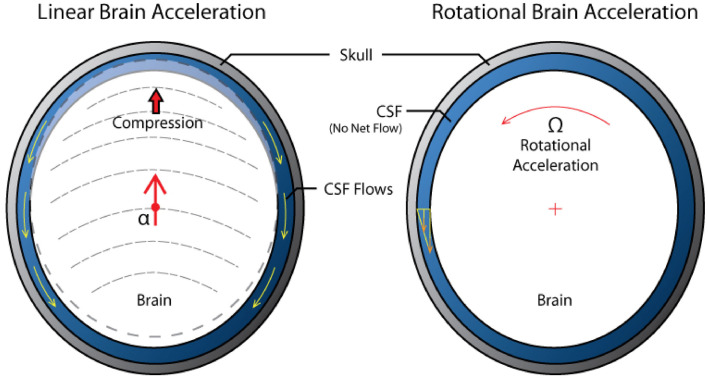
**Schematic of brain-CSF interaction in linear and rotational acceleration**.

The primary compression/tension and shear waves are followed by the macroscopic translational and rotational motion of the brain inside the cranium due to inertial forces. Combination of linear and, more importantly, angular accelerations of the brain often lead to diffuse axonal injury (DAI), contusion, and acute subdural hematoma (Smith and Meaney, 2000; King et al., 2003; Rowson et al., 2012). Experimental tests on human volunteers (Feng et al., 2010) and cadavers (Hardy et al., 2001; Zou et al., 2007) showed that even for low-severity impacts in the sagittal plane, the brain translation has a magnitude of 4–5 mm, and rotation is on the order of ±5 °and lasts for almost 300 ms. Due to the brain’s asymmetry and attachment to the brain stem, linear displacement of the skull leads to both linear and angular displacement of the brain relative to the skull. From the hydrodynamic point of view, there is a difference between translational and rotational brain movement in the skull, Figure 4. Linear translation of the brain within the skull induces compensatory volumetric flow of the CSF. On the other hand, the rotation of a brain does not involve CSF displacement and the brain rotation is only opposed by the brain surface-CSF shear force. In reality, brain rotations in the horizontal and frontal planes are limited by falx while in the sagittal plane they are limited only by the brain stem anchoring and by bridging veins. As the CSF is almost incompressible any macroscopic brain translation has to be associated with the flow of the displaced surrounding fluid. In fact, the CSF is not only responsible for the well-known neutral buoyancy of the brain, it also provides a “hydrodynamic lubrication” protection of the brain from contact with the cranium. It has been hypothesized that with sufficient forces however, the brain may impact the cranium at the impact point (coup injury) and at the opposite site (contrecoup injury) leading to laceration, contusion, or hemorrhage (Smith and Meaney, 2000). The relative linear inertial acceleration of a floating object in a closed volume is only possible if the density of the suspended object (brain) is different from the surrounding fluid (CSF). The brain-CSF density difference is very small (ρ_Brain_ ~1020 kg/m^3^, ρ_CSF_ ~1005 kg/m^3^) so the relative motion will be slow and it may take a long time for the brain to contact the cranium. This has been experimentally observed in a physical surrogate model of an idealized head (van den Akker, 2010). It should be pointed out that the rotational motion of the brain within the cranium is not constrained by the density difference, it can exhibit much higher velocities and, because of geometrical non-uniformity of the cranium, it may result in a brain-cranium contact. Experimental tests in animal models of TBI have shown that the rotational motion of the brain is much more damaging and can be responsible for focal and diffuse injuries, even in moderate and mild events leading to brain rotation relative to the skull (Margulies, 2000; King et al., 2003; Eucker et al., 2011). Maintaining the head in a rigid posture allowing linear but no rotational accelerations may explain why race car drivers have survived crashes of 50–80 g; and why woodpeckers can decelerate their brains up to 1200 g during prolonged wood pecking yet can be knocked unconscious by inadvertently flying head first into a window (May et al., 1979; Margulies, 2000).

As the brain is connected to the rest of the body through large blood vessels and the spinal canal, there is a strong possibility of the blast energy to enter the brain in the form of elastic waves propagating along the vessels to the brain (Bhattacharjee, 2008; Courtney and Courtney, 2009; Cernak, 2010). Because the vascular elastic wave speed is very low (10–15m/s), these waves will arrive at the brain after the initial blast. We define the *primary brain injury* as the mechanical damage to brain structures caused by the initial stress (pressure, shear) waves traversing the brain just after the blast impact and after any mechanical impulse to the head, all lasting tens to hundreds of milliseconds. Accordingly the *secondary brain injury and repair* involves a time evolving myriad of biophysical, neuro-biological, physiological, and potentially cognitive mechanisms, caused by the primary injury, and lasting for hours and, sometimes for life.

Current understanding of primary injury mechanisms to the brain microstructures is very limited partially because of anatomical heterogeneity of the brain, very short duration of injury events, difficulties in collecting *in vivo* experimental data from animal models, and lack of adequate mathematical models. It is clear, however, that part of the energy in waves traversing the brain is absorbed by various brain structures including vasculature, axonal tracks, neuronal dendrites and synapses, cytoskeleton, and ion channels causing localized and diffuse damage of the primary injury. From the mechanical perspective, macro and micro interfaces between structures with disparate properties (density, elastance) are particularly vulnerable to damage caused by high stain rate loads typically observed in the blast brain injury. These macro and micro interfaces may include the blood brain barrier (BBB), choroid plexus, brain-CSF interface, neuronal/axonal membranes, nodes of Ranvier, dendritic spines, synaptic clefts, transmembrane structures, and others. These interfaces may be particularly susceptible to mechanical damage if their resonant properties are matched to frequencies of the primary wave. In severe TBI, the primary mechanical damage, e.g., skull fractures or hematomas can be visibly detectable on CT or MRI images. In concussions and mTBI, the primary damage, such as diffuse neuroaxonal injury (Smith and Meaney, 2000; Stys, 2005; Pullarkat et al., 2006; Tsutsui and Stys, 2012), microvascular injury (Dietrich et al., 1994; Readnower et al., 2010; Chodobski et al., 2011), and synaptic injury (Albensi, 2001; Ferenc et al., 2009; Przekwas et al., 2009; Ding et al., 2011) are very difficult to detect, even with high resolution diffusion tensor images (Mac Donald et al., 2011).

### Secondary injury and repair mechanisms

**Figure 5:**
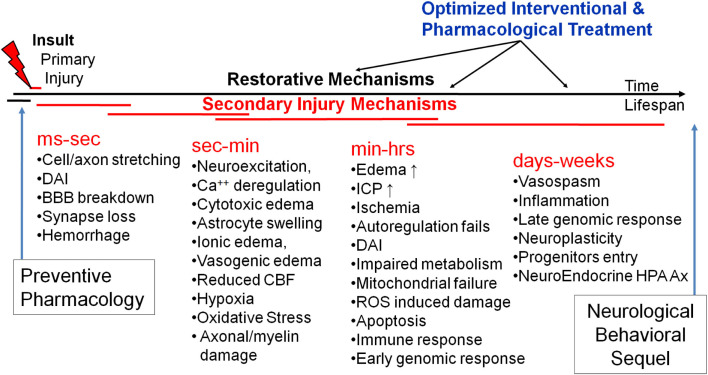
**A schematic of time-structured secondary brain injury mechanisms and potential windows for pharmacological intervention**.

The primary mechanical insult results in a cascade of secondary injury and repair mechanisms. *In vivo* and *in vitro* experimental models of TBI have begun to unravel the mechanisms producing secondary mechanisms (Kochanek et al., 2000; Smith and Meaney, 2000; Wieloch and Nikolich, 2006; Cernak, 2010; Barkhoudarian et al., 2011; Risling et al., 2011). Figure 5 schematically illustrates our attempt to establish a timeline of secondary mechanisms as well as windows for optimized pharmacological treatment. In general, they can be classified into several related categories including: biophysical, metabolic, neurochemical, and inflammatory. Each of them includes several mediators involved in a constellation of neuro-biological pathways, most of which are still poorly defined.

Immediately after a mild or moderate primary brain insult, significant electrochemical and hydraulic (osmotic) exchange of ionic, molecular, and fluid constituents occurs between the intracellular and extracellular neuronal and microvascular structures. This exchange generates cytotoxic and ionic swelling (edema) of astrocytes and neurons at the expense of the extracellular space, causing reduction in the diffusive transport of vital metabolites and neurotransmitters. The limited intrinsic energy reserves of the CNS require continuous supply of glucose (Glc) and oxygen for a normal function, most notably for neurotransmission (predominantly for glutamate (Glu) uptake) and for neuronal ionic repolarization (Ca^++^ and Na^+^/K^+^ exchange). Any additional energy requirements needed for structural repair of the injury will have to be facilitated by accelerated supply and utilization of Glc and oxygen to produce ATP. This hypermetabolic state may cause local cerebral ischemia, hypoxia, neuroexcitation, and failure of axonal and neuronal conduction. Because the intra-arterioral and capillary blood pressures are much larger than the intracranial pressure (ICP), any mechanical damage to the BBB may cause efflux of water and small molecules (some of them neurotoxic) from the luminal to the interstitial space causing brain swelling, mechanical deformation of the brain tissue and increase of ICP. These in turn may compress the venous and CSF volumes reducing the cerebral blood flow (CBF) rate and causing ischemia. Altered metabolic states may result in further cellular/mitochondrial damage due to oxidative stress, while penetrating blood borne molecules such as cytokines may initiate inflammatory responses (Schmidt et al., 2004; Graber and Dhib-Jalbut, 2009).

Diffuse axonal injuries are the hallmark of mild and moderate TBI and are caused by a combination of rapid tension and shear deformations in the white matter of the brain (Smith and Meaney, 2000; Stys, 2005; Johnson et al., 2012; Tang-Schomer et al., 2012). DAI is believed to be present in all mTBI injuries accompanied with a loss of consciousness (Meythaler et al., 2001), yet each year more than 1.5 million Americans sustain mTBI with no loss of consciousness and no need for hospitalization (DeKosky et al., 2010). The mechano-biological mechanisms of axonal injury are not well understood and are an active research area (Tsutsui and Stys, 2012). Mechanical reasoning indicates that large and rapid forces to an axonal bundle can cause primary axotomy, as in the breaking of a fiber in a rope under large tension, while other axons may only experience partial damage to some of its structures (membrane, cytoskeleton, ion channels). These “partially damaged” axons may further undergo complex and prolonged biophysical and metabolic responses (see secondary injury below) leading to either the axonal repair or cause an irreversible axonal damage, i.e., formation of retraction bulbs. It should be mentioned that secondary effects following non-mechanical injury, e.g., ischemia or neuro-inflammation can display some of the same traits as DAI (Tsutsui and Stys, 2012). The mechanical integrity of the axonal plasma membrane, a critical barrier between intra- and extra-cellular environments, is essential for neuronal function and survival. Even intermittent membrane mechanoporation may result in axonal electrical depolarization which may cause rapid electrochemical and osmotic “fluxing” of ions and water resulting in axonal swelling (Tang-Schomer et al., 2012). Mechanical forces may also disrupt a network of axonal cytoskeleton responsible for structural integrity and intra-axonal two way traffic of various cargo (Fernandez and Pullarkat, 2010). For the neuron to recover from these mechanical derailments it will have to initiate a metabolic “overdrive” (hypermetabolism) needed for electrochemical and osmotic repolarization, and membrane and cytoskeleton repair. Unfortunately, increased hypermetabolism can also be also damaging via oxidative stress.

Primary mechanical damage to axonal tracks, often present in the parasagittal white matter of the cerebral cortex, corpus callosum, and the brain stem, initiates a cascade of secondary axonal injury and repair mechanisms (Smith and Meaney, 2000; Stys, 2005; Pullarkat et al., 2006; Wieloch and Nikolich, 2006; Tang-Schomer et al., 2012; Tsutsui and Stys, 2012). Axonal fibers with damaged myelin and plasma membrane suffer large current leaks and exhibit increased metabolic requirements to support conduction. Action potential propagation under these conditions exacts a high metabolic price for energy-consuming ion movements, which in turn places increased demands on energy-consuming Na–K ATPase ion exchangers. This, combined with a potentially impaired metabolic ability of mitochondria, may produce a state of chronic axonal hypoxia, deregulation of Ca^++^ homeostasis and ultimately structural failure of the fiber, manifested as spheroid formation and finally transection in the form of microbeads, retraction bulb, and axonal transection formation (Stys, 2005; Kilinc et al., 2009; Tang-Schomer et al., 2012).

Diffuse synaptic and dendritic spine injuries are also potentially significant secondary injury and repair sites. These TBI mechanisms have not been reported in the open literature, probably because of lack of viable *in vivo* experimental measurement modalities at such small scales. Synapses are tiny structures (~1 μm in diameter and ~20-nm spacing) precisely packed in the CNS at an incredibly high density (estimates range from ~ 2 × 10^8^ to 4 × 10^9^ in rat’s brains) (McAllister, 2007). Synaptic terminals are mechanically very dense structures composed of a remarkably large number of proteins, transsynaptic adhesion molecules, and scaffolding. Its proper function strongly depends on its morphology because mechanical deformations may cause malfunction. It is likely that mechanical tension and shear waves cause temporary disconnects and microdamage of synapses and dendritic spines which in turn result in temporary cognitive malfunction (Monnerie et al., 2010; Gao et al., 2011). It is also likely that a large number of deformed synapses in mild injury may be “repaired” by electrokinetic and biomechanical mechanisms – a process of synaptic neuroplasticity and cognitive recovery. The proposed mechanism of synaptic injury has been recently observed in *in vitro* neuronal cultures (Ferenc et al., 2009; Monnerie et al., 2010) exposed to shock waves. These results suggest that shockwaves emanating from explosive devices may specifically affect synaptic plasticity in the brain. Further, *in vitro* and *in vivo* experiments and mathematical modeling studies should be conducted to elucidate these injury mechanisms and to determine whether the diffuse synaptic injury plays a prominent etiological role in mTBI.

## Multiscale, Multi-Discipline Modeling of Blast TBI

### Multiscale model of blast TBI – overall approach

A comprehensive computational model of blast TBI should involve several disciplines including: blast wave gas dynamics, human body dynamics, body/head/brain biomechanics, physiological responses, and a host of biophysical and neuro-biological mechanisms of secondary injury and repair. The complexity of a mathematical model of blast wave TBI is magnified by a wide spectrum of length and time scales:

- Length – from meters for a blast scene, to centimeters for the brain, to micrometer for neurons and axons, to nanometer for neuronal synapses
- Time – from microsecond for blast wave transition over the head, to millisecond for brain biomechanical responses, to min/h/days for secondary injury and repair cascade.

To develop such a mathematical model of TBI, a coordinated effort is needed integrating various disciplines including: neuroimaging, neuroanatomy, geometry/mesh generation, computational fluid dynamics (CFD), finite element method (FEM) structures, biomechanics, and computational neurophysiology and neurobiology. A coupling between primary injury models (biomechanical) and the secondary mechanism models (neurobiology) is also required. Development of complex and computationally expensive high-fidelity 3D models for a human and an animal (rat, pig) should be accompanied with the development of “reduced” (compact) but computationally fast models using approximate anatomic/geometric representation, yet affording advanced models of neurobiology. The model development effort should be paralleled with model-guided experiments on neural cells and brain tissue cultures, animal models, physical surrogates, and to some extent on humans to generate benchmark quality data for model validation and scaling.

**Figure 6:**
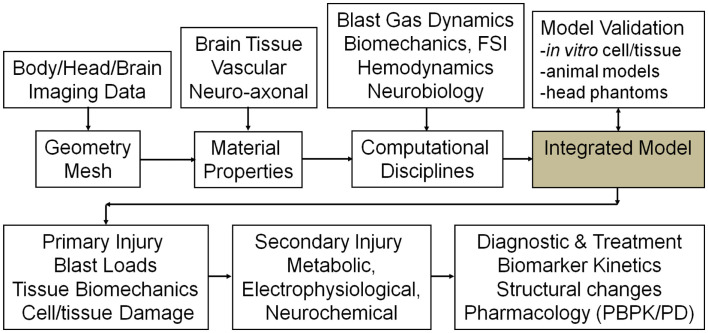
**Schematic of the simulation framework, tools and interfaces, and expected results**.

Computational models of blast wave physics and human body/head/brain biomechanics, have been developed over the last few decades for military/aerospace and automotive safety applications (Takhounts et al., 2003, 2008; Anderson, 2004; Kleiven, 2007; Horgan and Gilchrist, 2008; Needham, 2010; Zhang et al., 2011). For modeling blast TBI, further improvements are needed in: high strain rate tissue material properties, coupled fluid-structures interaction (FSI) of intracranial biomechanics, micromechanics of brain tissue damage, and coupling between the brain macro- and micro-scale biomechanics. Computational models of brain secondary injury and repair mechanisms have not been established yet, mainly because of complexity and incomplete understanding of the processes involved and partially because of lack of supporting benchmark quality *in vitro* and *in vivo* experimental data. There are however, several mathematical models of neurophysiology and neurobiology which could be used as a starting point for the development of a comprehensive model of the secondary mechanisms (Cooley and Dodge, 1966; Koch and Segev, 1998; Ursino et al., 2000; Lakin et al., 2003; Wakeland and Goldstein, 2005; Ascoli, 2006; Carnevale and Hines, 2006; Aubert et al., 2007; Gleeson et al., 2007; Humphrey et al., 2007; Savtchenko and Rusakov, 2007; Cloutier et al., 2009; Linninger et al., 2009; Mangia et al., 2009; Kozloski and Wagner, 2011; Liang et al., 2011; Mohan et al., 2011). The schematic in Figure 6 shows a potential functional layout of such a modeling platform. It could set the standard for comparison of alternative model components and establish a benchmark framework for model calibration and validation against experimental data. Following is a brief overview of key model components, existing models in selected disciplines and suggestions for further development.

### Multiscale model of primary TBI

#### Anatomy/geometry/mesh

Accurate simulations of blast wave interaction with a human or animal body requires 3D anatomical/geometric models that could be used to generate computational meshes for CFD and FEM biomechanics models. Anatomical models can be generated using neuroimaging data of brain and skull structures and the whole body imaging data, e.g., “Visible Human” (Spitzer and Whitlock, 1998; Spitzer and Scherzinger, 2006; Tang et al., 2010). Reasonable resolution models of a rat and mouse whole body anatomic geometries are also available (Segars et al., 2004; Bai et al., 2006; Khmelinskii et al., 2011). Improved resolutions of rodent brain functional zones as well as body/cerebral vascular anatomy have to be established. The anatomic geometry models are used to generate computational meshes outside and inside the body for blast and biomechanics simulations. To simulate whole body bio-dynamics (movement in air induced by blast loads) the anatomical/geometric models need to be “articulated,” i.e., individual body parts should be connected by joints to enable their relative motion (Wilkerson and Przekwas, 2007; Arepally et al., 2008; Zhou and Przekwas, 2011; Tan et al., 2012).

Several anatomical/geometry models have been developed to study human body/head impact injury biomechanics (Zhang et al., 2001; Levchakov et al., 2006; Mao et al., 2006, 2010; Kleiven, 2007; Horgan and Gilchrist, 2008; Ramirez, 2010; Gayzik et al., 2011; Yasuki, 2011) and rat head and body injury (Liang et al., 2011; Przekwas et al., 2011). Until recently brain injury models focused on the inertial and impact (crash) injury for which the skull and brain geometry were sufficient. Because the blast loads are spatially and temporally distributed over the entire head and neck, the anatomical model should include the head’s skin, facial structures including ocular and nasal cavities, cranium, and neck geometry. Such anatomical geometries are currently being developed (Chafi et al., 2009; Moore et al., 2009; Przekwas et al., 2009, 2011; Nyein et al., 2010; Ortega, 2011; Zhang et al., 2011; Sundaramurthy et al., 2012). Since blast loads occur at very fast rates, the brain injuries tend to be spatially distributed loci of micro-injuries, e.g., DAI. The current models of whole brain biomechanics do not have the proper resolution to model the micro-scale injuries that result from a primary blast exposure. Furthermore, to the best of our knowledge, none of the existing models can properly simulate the physics of the brain-CSF interaction or the head-neck movement.

Experimental *in vitro* tests of brain tissue slices and neuronal cell cultures may enable validation of mathematical models by providing detailed correlation between the primary injury dynamics and the resultant secondary mechanisms (Morrison et al., 2006, 2011; Chen et al., 2009; Frieboes and Gupta, 2009; Yu and Morrison, 2010; Johnson et al., 2012; Tang-Schomer et al., 2012). It is important to develop protocols and tools for the generation of 3D morphological geometries of *in vitro* cell and tissue cultures and their dynamic responses to mechanical or shock wave loads. Ideally, such models should register mechanical, electrokinetic, and biochemical spatiotemporal responses of axonal, synaptic and sub-cellular structures to controlled mechanical insults.

#### Blast wave gas dynamics and intracerebral fluid mechanics

**Figure 7:**
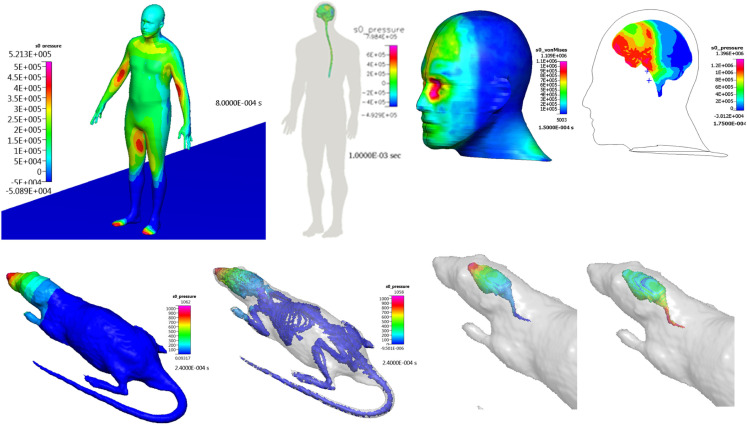
**3D Anatomical/geometric models of a human body and head, and a virtual rat**.

Computational fluid dynamics models have been successfully used to simulate blast wave dynamics over a human body and head (Imielinska et al., 2006; Przekwas, 2008; Moore et al., 2009; Taylor and Ford, 2009; Needham et al., 2011) and to calculate pressure and shear forces for subsequent modeling of human body biodynamic and biomechanical responses. Figure 7 shows examples of CFD model predictions of blast wave interaction with a human body, head, and with a rat body. Reported simulations have shown that such a sequential modeling approach is well justified as the inertial body movement starts well after the blast wave traverses the body (Needham et al., 2011; Tan and Przekwas, 2011). Accurate simulation of moving shock waves and their interaction with solid objects without “smearing” of the shock front discontinuities requires small time steps, very fine computational mesh in the entire flow domain and long computing times. Fine mesh is essentially only needed in the regions of high gradients, e.g., shock front, and much coarser grids could be used elsewhere. One way to solve this problem is to use a solution adaptive mesh refinement.

Computational fluid dynamics models could also be used to simulate the responses of intracranial fluids, including CSF-brain interaction and cerebral blood interaction with brain tissue. Since the movement of intracranial fluids is strongly coupled to mechanical displacements of the skull and the brain, the intracranial fluids should be simulated using a FSI model. Direct numerical simulations of the FSI in a closed intracranial cavity is a non-linear and computationally very challenging problem, as small cranial displacements cause large variations in the ICP. To the best of our knowledge, the intracranial FSI problem has not been convincingly solved, yet. Reported simulation results exhibit negative absolute pressures (Moore et al., 2009; Moss et al., 2009; Taylor and Ford, 2009; Przekwas et al., 2011), trap predicted fluid pressures to a prescribed value (e.g., absolute zero or vapor saturation) or show large cavitation volumes (Wardlaw and Goeller, 2010). In most reported models, CSF is treated as compressible deforming “solid” attached to brain and skull, an assumption valid only for the first few millisecond when there is no significant flow of CSF. However, if longer time scales need to be simulated, such as for shear waves, brain rotation, and brain swelling during edema, a full FSI model may have to be used. Such an approach has been used for high fidelity and reduced order modeling of hydrocephalus (Kurtcuoglu et al., 2007) but has not been well established in 3D TBI models, yet.

**Figure 8:**
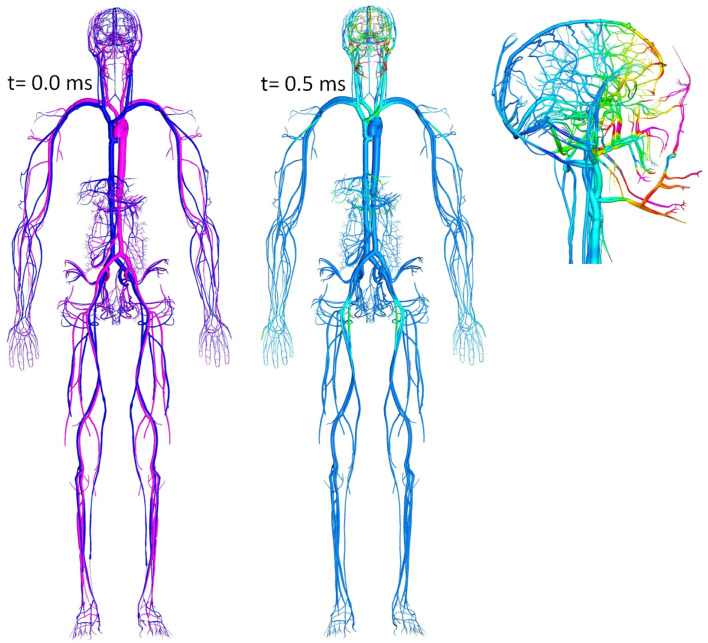
**Whole body cardiovascular system model “embedded” in the tissue biomechanics model used to study blast-induced elastic waves**.

An FSI model, coupling whole body biomechanics, elasto-fluid dynamics of thoracic/cerebral vascular system, and brain biomechanics could be used to evaluate the “thoracic” or “vascular” hypothesis of TBI (Cernak et al., 2001; Chavko et al., 2011). This hypothesis states that a thoracic/abdominal vascular system, compressed by a blast wave, may induce an elastic wave propagation from the thorax along the vascular system to the brain. This may cause brain tissue damage and BBB injury. Since vascular elastic waves propagate relatively slowly (12–15 m/s), these injury events occur much later than primary blast events. Figure 8 shows a computational model of a human vascular system coupled to the body/brain biomechanics, currently under development to evaluate the above hypothesis (Przekwas et al., 2011).

An FSI model may be also required to study brain-vascular coupling during vasogenic edema, hemorrhage, and vasospasm, all associated with brain injury (Armonda et al., 2006; Armin et al., 2008; Alford et al., 2012). Mechanical microdamage to the BBB causes an increase of vascular permeability and inflow of osmoles and water to the brain causing volumetric expansion of the brain and increase of the ICP – a consequence of the so-called Monroe–Kellie doctrine. This in turn results in compression of the vascular (venous in particular) system and potentially brain herniation. In such a case, mathematical models of brain poro-visco-elastic biomechanics need to be coupled to CSF flow, vascular fluid mechanics as well as to electrochemistry of solutes and ion transport and osmotic pressure developments.

#### Head and brain biomechanics

Computational modeling of human head injury biomechanics has been investigated since the 1970s, first using approximate analytical and spring-mass-damper (SMD) models (Slattenschek and Tauffkirchen, 1970; Alem, 1974) and in 1990s using FEM (Ruan et al., 1993; King et al., 1995). Today FEM tools are routinely used to simulate impact biomechanics and primary brain injury problems, particularly in the automotive occupant safety applications (Miller, 2011). Advanced 3D FEM models of head/brain anatomy and biomechanics and injury have been pioneered at the Wayne State University resulting in the well-known WSUBIM (Wayne State University Brain Injury Model) FEM human head model (Ruan et al., 1993; Zhou et al., 1996; Al-Bsharat et al., 1999). This model of a 50th percentile male human head currently includes scalp, cranium, falx cerebri, tentorium, sagittal sinus, transverse sinus, bridging veins, CSF, and the brain structures as separate anatomical segments (Zhang et al., 2001, 2011; Hu et al., 2007). Svein Kleiven’s group at the Royal Institute of Technology in Stockholm, Sweden has developed a human head/neck FEM model with improved resolution of the subarachnoid CSF and 11 pairs of parasagittal bridging veins (Ho and Kleiven, 2007; Kleiven, 2007). A neck model including spinal column, spinal cord, dura mater, and neck muscles was incorporated allowing the brain stem to be extended to the spinal cord. Other FEM head/brain biomechanics and injury models include: Simulated Injury Monitor (SIMon) FEM human head model developed by a team lead by Takhounts at the National Highway Traffic Safety Administration (NHTSA) (Takhounts et al., 2003, 2008), the University College Dublin Brain Trauma Model (UCDBTM) (Horgan and Gilchrist, 2008; Colgan et al., 2010), and the Strasbourg University Finite Element Head Model (SUFEHM) (Willinger et al., 1999; Raul et al., 2008; Meyer et al., 2013) as well as others. All of these models, in spite of successes in modeling head impact and inertial translation/rotation accelerations, still need improvements in anatomical geometry, physics, and numerics, e.g., high strain rate material properties, modeling the CSF flows, accounting for the presence of vasculature, adequately model the micro-scale injuries, addressing numerical stiffness, and long computing times.

**Figure 9:**
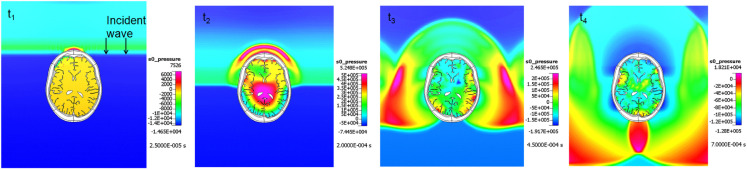
**Example coupled CFD-FEM simulation results of a blast wave diffraction around, and transmission through a human head**. A sequence of four time instances.

In the last few years, FEM head/brain biomechanics models have been adapted for modeling the blast TBI by incorporating head/face anatomical details and by coupling them to the blast physics CFD solvers (Ziejewski et al., 2007; Mott et al., 2008; Przekwas, 2008; Chafi et al., 2009, 2010; Moore et al., 2009; Moss et al., 2009; Przekwas et al., 2009, 2011; Taylor and Ford, 2009; Nyein et al., 2010; Zhang et al., 2011; Panzer et al., 2012a; Zhu et al., 2013). Figure 9 presents example simulation results of a shock wave reflection from and diffraction around a human head (coronal cross section) and a resultant pressure wave within the brain. In comparison to the blunt brain biomechanics model, the blast injury model has a loading force that is much faster and is spatially and temporally “distributed” over the entire head during the shock wave propagation around the head. Moreover, the intracranial loads, both compression and tension, and the strain rates are much higher in the blast case. Coupled blast wave gas dynamics and brain biomechanics simulations are needed to compute dynamic response of the head, cranium, and the brain.

The main limitation of all existing FEM models is the treatment of the CSF interaction with the brain and the cranium, particularly modeling the shear waves and brain translation/rotation. Most of the FEM models treat the CSF as a “solid with fluid-like property” allowing a “contact with slip” interfaces between CSF and skull and brain. This approach is inadequate for modeling larger deformations and for modeling brain translation and rotation for longer periods of time. For short duration head/brain primary biomechanical events, lasting only tens of milliseconds, typically the explicit FEM models are used to simulate brain responses. However for longer duration events, such as propagation shear waves, brain rotation, swelling, and CSF displacement, implicit FEM schemes are required. These however require full matrix inversion and are much more difficult to solve for fine meshes, even using parallel computers.

Another very important and challenging problem is the development of material properties for the skull and various anatomical regions of the brain for high strain rates, typical in blast loads. It is clear that different head/brain tissues will require tissue-specific constitutive equations and parameterization. In spite of decades of experimental testing and analytical studies of brain mechanical properties, no universally accepted dataset exists and the material property parameters vary by an order of magnitude (Hrapko et al., 2008). Material models have been extracted from both *in vitro* experimental data (Takhounts et al., 2003; Brands et al., 2004; Miller, 2011; Prevost et al., 2011) as well as from *in vivo* data (Gefen and Margulies, 2004; Atay et al., 2008; Clayton et al., 2011). The results of different studies are difficult to compare, due to the wide range of experimental protocols including the species type/age (human, rodent, porcine), loading configurations (compression, tension, shear, indentation), the loading histories (cyclic, stress relaxation creep), and test regime (levels of strains and strain rates, temperature, tissue hydration). The experimental data have facilitated the development of a large variety of constitutive models ranging from simple linear elastic, hyperelastic, linear viscoelastic to non-linear viscoelastic, yet no consensus exists even on the linear viscoelastic properties. No model has integrated viscoelastic, stress relaxation, and large strain response into one single constitutive framework thus far. Before significant advancements can be made, the modeling and testing communities must come to a consensus on the material model formulations and anatomical/geometric and numerical representations of the FEM head/brain biomechanics and injury model.

Finite element method biomechanics models may also be used to simulate brain tissue/cell damage at the micro-scale. Mathematical models of mechanical damage to neuroaxonal structures may be able to describe damage to cell membranes, cytoskeleton, ion channels, synaptic clefts, dendrites, and axons. These in turn, could provide inputs for the secondary injury and repair models, simulating electrophysiology and ion homeostasis, alterations in metabolism, neuroexcitation, cytotoxic edema, oxidative stress, apoptosis, and other injury and repair mechanisms. In the last few years, the first FEM biomechanics simulations of very simplified axonal structures have been reported (Karami et al., 2009; Cloots et al., 2010; Przekwas et al., 2012). Future advancements in micro-scale FEM can incorporate boundary conditions from macro-scale simulations (Przekwas, 2008; Cloots et al., 2010; LaPlaca and Prado, 2010). *In vivo* micro-imaging may also provide functional response data (electrophysiological, metabolic, and biochemical) needed for the development and validation of mathematical models of secondary brain injury and repair mechanisms. We envision that the next generation of *in vivo* and *in vitro* micro-biomechanics models will be able to elucidate neuroaxonal injury mechanisms and will help establish brain region and insult specific injury criteria.

### Multiscale model of secondary injury and repair mechanisms

The secondary brain injury and repair mechanisms start immediately after the primary insult and, depending on the injury severity, may last for a long period of time (Graham et al., 2000; Margulies, 2000; Cernak et al., 2001; Cernak, 2010; Masel and DeWitt, 2010; Meaney and Smith, 2011). Secondary mechanisms are multiple, interacting cascades of local and systemic responses. Although primary injury comprises the initial tear and shear of neuro-tissue, secondary mechanisms can dramatically exacerbate the initial injury, or conversely, participate in neuro-repair processes.

Development of a mathematical model integrating all secondary mechanisms is a formidable task. A mathematical model of secondary brain injury and repair that couples biomechanics, cerebral perfusion, brain metabolism, and neurobiology does not exist yet. At the same time several components of such a model have been developed and reported including: cerebral perfusion, fluid electrolyte balance, metabolism, cellular signaling pathways, electrophysiology, edema, neuroexcitation, etc. (Yi et al., 2003; Wakeland and Goldstein, 2005; Dronne et al., 2006; Gleeson et al., 2007; Humphrey et al., 2007; Linninger et al., 2009; Østby et al., 2009; Mohan et al., 2011). Traditionally, computational neurophysiology and systems biology have been evolving as separate disciplines and only recently has it become clear that their combination may enable revolutionary progress in neurology (De Schutter, 2008). Cerebral physiology, neurobiology, and secondary injury models are typically formulated using a multi-compartmental modeling approach linking cerebral vascular, interstitial, and intracellular compartments. The next generation secondary brain injury and repair models will have to combine compartmental or distributed models for the *in vivo* whole brain physiology coupled to neuroaxonal and synaptic biophysics and neurobiology models. From the brain injury modeling perspective it will be essential to combine models of biomechanics and neurobiology, validate them on *in vitro* experiments, and evaluate them on *in vivo* animal/human data.

#### Models of head/brain biomechanics and cerebral hemodynamics

Mathematical modeling of brain biomechanics can be accomplished using both FEM models as well as much simpler but computationally efficient SMD elements. SMDs can be adapted for modeling both macroscopic biomechanical effects of secondary mechanisms, such as cerebral arterial/venous elasticity, hemorrhage, edema and vasospasm, as well as microscopic biomechanics of brain cell/tissue injury, e.g., BBB breakdown, axonal and synaptic injury (Di Bona et al., 2003). The mechanical model will have to be coupled to models of CBF, brain perfusion, and volume shifts between brain compartments. Similar SMD modeling approach has been used for modeling blast lung injury (Przekwas, 2008; Stuhmiller, 2008).

**Figure 10:**
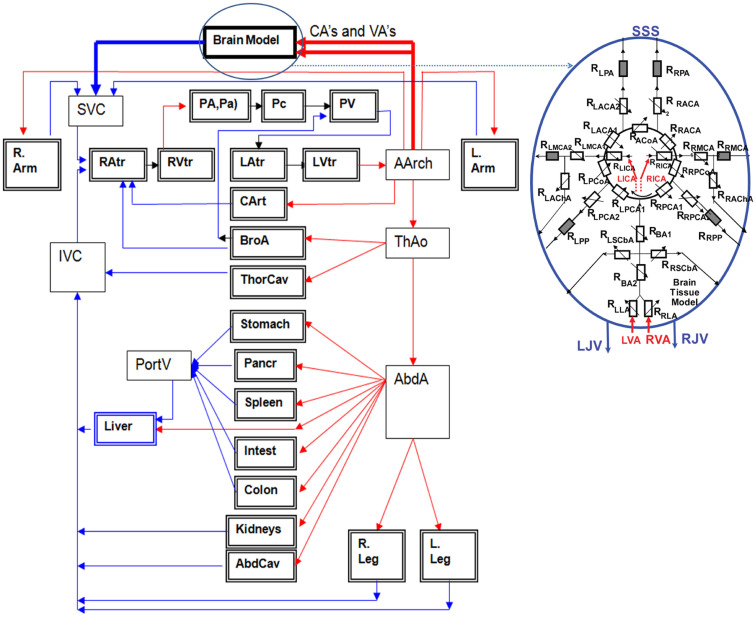
**Whole body multi-compartmental human cardiovascular system and the cerebral perfusion (circle of Willis) models**.

Reduced order fluid-network models have been used for modeling CBF and tissue perfusion, autoregulation, and other aspects of cerebral physiology (Ursino et al., 2000; Wakeland and Goldstein, 2005; Alastruey et al., 2007; Stevens et al., 2008; Linninger et al., 2009; Liang et al., 2011). More elaborate models use networks of blood vessels arranged to represent the topology of the circle of Willis (COW) connected to the whole body circulation (Reymond, 2011). These models solve for time/space resolved intracranial blood flow rate, pressure, and fluid/metabolite exchange between vascular and brain tissue compartments. To simulate brain injury the cerebral hemodynamics model may need to be coupled to a brain biomechanical model via transmural pressure. Figure 10 shows examples of spatially distributed and multi-compartmental models of the human body, cerebral vascular system, arterial COW and anatomically distributed cerebral perfusion and venous return (Przekwas et al., 2011). In that model, the vascular tree can dynamically adjust its vessel radius to accommodate temporal changes in the perfusion pressure and autoregulation as well as changes in the ICP due to mechanical loads (e.g., blast wave). Ultimately, the cerebral vascular model should provide inputs to several other sub-models such as ischemia, hemorrhage, edema, hypoxia, vasoregulation, vasospasm, and a full range of neurobiology models. Combining a spatially distributed whole body/brain-vascular system model with the FEM body/head biomechanics models may help in elucidating the thoracic/vascular TBI hypothesis.

#### Cerebral metabolism and injury neurobiology models

Secondary brain injury and repair is a multi-factorial process involving a range of bio-electro-chemical events, but two components are of key importance – alterations in metabolism and neurotransmission (Rzigalinski et al., 1998; Magistretti and Pellerin, 1999; Aubert et al., 2007; Payne et al., 2009; Cernak and Noble-Haeusslein, 2010; Peskind et al., 2011). Mathematical models of neurometabolic mechanisms are typically derived from kinetic pathways, involving a large number of kinetic parameters obtained from *in vitro* experiments (Nicholson, 2001; Banaji et al., 2005; Qutub and Hunt, 2005; Dronne et al., 2006; Descombes and Dumont, 2008; Payne et al., 2009; Orlowski et al., 2011). Some of these models already include key spatial compartments (vascular, interstitial, glial, neuroaxonal, and synaptic), incorporating several metabolic steps and are often linked to a glutamate-glutamine cycle, the key element of neurotransmission and neuroexcitation. As discussed in Section “Secondary Injury and Repair Mechanisms,” the axonal potential propagation and synaptic neurotransmission consume the majority of the available metabolic energy, so perturbations of the energy supply may affect the function of individual neurons and their network. An integrated model of bio-energetics should combine models of tissue perfusion, energy metabolism, related oxidative stress and neurotransmission. In the last few years integrated models of glucose-lactate (Glc-Lac) energy metabolism, synaptic neurotransmission and the neuron-astrocyte glutamate-glutamine (Glu-Gln) cycle have been reported (Aubert et al., 2007; Cloutier et al., 2009; Przekwas et al., 2009). Such models should be able to simulate brain metabolic responses to increased permeability of the injured BBB and show changes in the intracranial volumes and pressure due to cytotoxic and vasogenic edema. This in turn may cause compression of the vascular system, reduction of the blood flow and development of ischemic and hypoxic regions.

#### Neuronal, axonal, and synaptic neurobiology and injury models

Mechanical damage and metabolic impairments have direct impact at the cellular level. Mathematical models of neuroaxonal and synaptic mechanobiology can provide a framework for better understanding of secondary injury and repair mechanisms. The micro-biomechanical model should capture the biphasic elasto-visco-plastic cellular response; the initial rapid primary damage to synaptic clefts, axonal membrane or BBB, followed by a slow mechanical recoil and recovery (e.g., membrane sealing, synaptic reconnection/plasticity, remyelination). The micromechanical model could be coupled to cellular electrophysiology and neurobiology models to simulate various secondary events such as solute, electrolytes and water shifts, cellular depolarization, local cytotoxic edema, initial hypermetabolism needed for repolarization, loss of action potential signals due to damage to ion channels, and current leaks, loss of synaptic transmission due to spillover of neurotransmitters outside of the synaptic cleft, synaptic plasticity (LPT, LDT), and other mechanisms. The results of such a model may be able to show that the exacerbated secondary mechanisms, not just the initial mechanical injury, may be responsible for the long-term neurocognitive effects. This in turn could identify targets for neuro-interventions and optimal treatment strategies. The development and validation of such models will require detailed spatiotemporal experimental data from *in vitro* neuroaxonal cell/tissue cultures, *in vivo* animal brain injury models and ultimately *in vivo* conditions in animals and humans.

Mathematical modeling of coupled micro-biomechanics and electrophysiology of neuronal injury has not been reported yet, but has been identified as an important recommendation (LaPlaca and Prado, 2010). Mathematical models of cellular electrophysiology have been developed and used for modeling single neurons and large neuronal networks (Kager et al., 2000; Calvetti and Somersalo, 2011; Kozloski and Wagner, 2011). Typically, mathematical models of neurons, such as neuron and genesis, combine the electrical cable theory for modeling action potential propagation and the Hodgkin–Huxley model to simulate ionic fluxes (Bhalla, 1998; Carnevale and Hines, 2006). Neuron models have been successfully used for a wide range of problems including detailed neurophysiology of complex 3D neurons, propagation of action potentials in myelinated axons and in neuronal synapses (De Schutter and Bower, 1994; Gleeson et al., 2007; Savtchenko and Rusakov, 2007; Lopreore et al., 2008; Brown et al., 2011; Kozloski and Wagner, 2011; Mohan et al., 2011). Significant progress has been achieved in establishing an infrastructure of experimental databanks of 3D neuronal morphologies and neurobiology data that could be used for the development and validation of mathematical models (Ascoli, 2006; Eberhard et al., 2006; Gleeson et al., 2007; Martone et al., 2008; He and Cline, 2011; Halchenko and Hanke, 2012; Leergaard et al., 2012).

**Figure 11:**
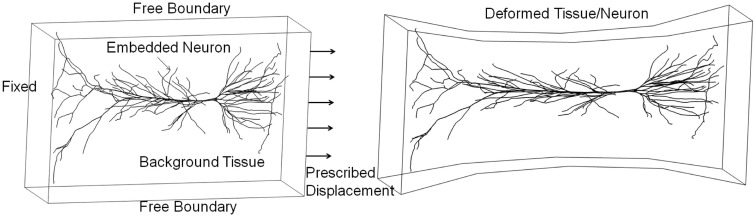
**Integrated biomechanical, electrokinetic, and metabolic model of an *in vitro* neuron exposed to mechanical stretching**.

An integrated micro-biomechanics and electrophysiology model could be developed based on *in vitro* neuronal cell/tissue cultures with well-defined mechanical loads and spatiotemporal measurements of cellular electrophysiological and biological responses (LaPlaca et al., 1995; Rzigalinski et al., 1998; Morrison et al., 2006, 2011; Lauret et al., 2009). This model could be validated on benchmark quality *in vitro* data, and then used to study *in vivo* neuroaxonal responses to brain injury loads. Figure 11 presents an example of a 3D neuron model “embedded” in a tissue culture exposed to mechanical stretch injury. In this test simulation, the FEM model of the tissue is coupled to a biomechanical-electrokinetic model of a neuron. The model simulates changes in the action potential propagation and metabolic support of axonal repolarization in response to mechanical damage to the neuroaxonal membrane.

## Model Validation Challenges and Opportunities

### Model validation and testing approach

The next generation TBI modeling framework will integrate several components, some already well established, e.g., CFD gas dynamics or FEM biomechanics, while others, such as tissue damage and the neurobiology of cellular injury, will have to be developed and validated. Among several challenges impeding the development of such a modeling framework are: incomplete understanding of injury mechanisms, limitations of existing computational tools in solving multiscale/multiphysics problems, and lack of benchmark quality test problems and experimental data for model validation.

**Figure 12:**
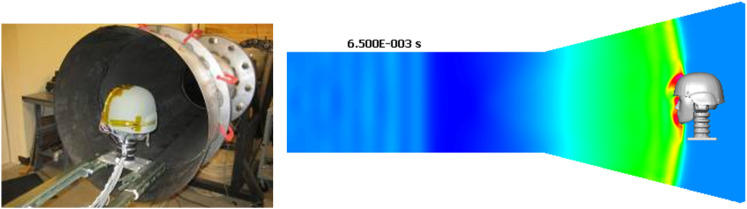
**A human head phantom in the shock tube for testing blast wave loading (Przekwas et al., 2011)**.

In the last few years experimental data directly related to the blast wave head/brain biomechanics and injury have started to emerge and could be used for model validation. To replicate the free-field blast wave loading in laboratory conditions, test articles such as head phantoms, animals (rats, mice, pigs), or cells/tissues have been placed inside or in front of a shock tube (Bayly et al., 2008; Säljö et al., 2008, 2011; Alley et al., 2011; Chavko et al., 2011; Leonardi et al., 2011; Risling et al., 2011; Shoge et al., 2011; Varas et al., 2011; Risling and Davidsson, 2012; Zhu et al., 2013). Shock tubes have been designed to control the pressure-time profile and impulse that replicate desired blast wave parameters (Reneer et al., 2011; Ritzel et al., 2011; Varas et al., 2011). Compared to round shock tubes, newer designs with square cross section allow better visual access to the test article (Sundaramurthy et al., 2012). Figure 12 shows a round shock tube with a conical exit section for testing a human head phantom with a helmet and hearing protection devices (Przekwas et al., 2012). A conical exit section not only allows more space for the test article, but also allows the formation of a spherical shock wave front resembling a free-field blast wave. It is also important to fully “expand” the wave to ensure a blast wave (Friedlander type) pressure profile.

#### Validation using head phantoms, cadavers, and humans

To validate the primary blast impact model it would be beneficial to establish benchmark anatomic/geometrical and material property models of selected physical phantoms and human (cadaver) heads. Several teams have used the HYBRID III head/neck phantoms as well as custom human head phantoms to evaluate its mechanical responses to shock tube loads (Bir et al., 2011; Leonardi et al., 2011; Qidwai et al., 2011; Varas et al., 2011; Goeller et al., 2012; Przekwas et al., 2012). Shock tube tests on a more anatomically accurate human head/neck phantom with soft skin-like features, cranial bone and brain tissue could become the basic validation test suite. The test procedures and experimental data should be fully documented to ensure proper setup of computational models, e.g., completeness of boundary conditions, locations of instrumentation sensors, etc. From the model development perspective, head phantoms offer several advantages including well-defined anatomical geometry, material properties, and sensor locations as well as reproducibility of tests and modularity of phantom setup, e.g., rigid vs. flexible neck. The benchmark data should include the free stream shock tube pressure traces (total and static) and skin pressures at several locations on the head and neck. If a brain surrogate is used, additional data should include ICPs at selected locations, preferably along three axes relative to the blast direction. Minimally intrusive time-accurate measurements of head and brain displacements at several locations could be used directly to compute strains, strain rates, tissue velocities and accelerations.

Experimental impact tests on human cadaver heads (Nahum et al., 1977; Hardy et al., 2001; Bir et al., 2011) have been used as benchmark data for the validation of FEM models. Cadaver tests are much more challenging since it is difficult to generate precise anatomical head and brain geometry of a specific specimen and to recreate physiological conditions, e.g., vascular perfusion, water-tight CSF space, etc. Compared to a live human, the surrogate models have serious limitations including inadequate anatomy and geometry, reproducibility, inadequate tissue properties, tissue decay in cadavers, and lack of physiology. In the last few years a new imaging technique, tagged MRI synchronized to periodic mechanical excitation has been developed for measuring mechanical deformations of human brain *in vivo* (Bayly et al., 2005; Atay et al., 2008; Sabet et al., 2008; Feng et al., 2010). It has been used to measure time/space accurate deformations of a human brain in response to rotational and translational deformations as well as loud sound waves in live human volunteers. The spatiotemporal fields of brain deformations and derived strains and strain rates could be used for the validation of a human head/brain biomechanics for non-injurious loads.

#### Validation on animal models

Direct experimental evaluation of *in vivo* brain injury is only possible using animal models (Cernak, 2005; Thompson et al., 2005), including rats (Dixon et al., 1987; Marmarou et al., 1994; Bayly et al., 2006; Chavko et al., 2007; Long et al., 2009; Bolander et al., 2011), mice (Carbonell et al., 1998; Cernak and Noble-Haeusslein, 2010; Rubovitch et al., 2011), pigs (Smith et al., 1997; Säljö et al., 2008; Bauman et al., 2009), and primates (Lu et al., 2012). Experimental tests on animal models provide a correlation between known insult level to injury response measured by taking samples from the brain for histochemistry analysis or by behavioral tests. Traditionally, animal models of TBI were developed to reproduce impact or acceleration loads such as the controlled cortical impact (CCI), the fluid percussion injury (FPI), and head acceleration and rotational models (Cernak, 2005; Morrison et al., 2011). Because the CCI and LFP require craniotomy and cause focal injury, they are not suitable to study blast brain injury, which typically is a closed head, diffuse type injury. Similar to head models, to better represent the blast brain injury in the open field, several teams have exposed animals, including rats and pigs, to shock waves generated by various types of shock tubes (Chavko et al., 2007; Bauman et al., 2009; Long et al., 2009; Risling et al., 2011; Sundaramurthy et al., 2012). In spite of ongoing challenges with animal position, orientation, and immobilization in the shock tube a method of scaling the shock wave pressure profile to an equivalent human dose needs to be developed. Shock tube animal tests may be able to reproduce DAI representative of human mTBI, reveal the role of head/neck movement in blast brain injury, and provide valuable data for the development and validation of mathematical models of mTBI. Recent experimental tests of mice and primates directly exposed to open field explosives detonation may provide additional information for calibration of shock tube models (Rubovitch et al., 2011; Lu et al., 2012). The shock tube animal tests provide the intracranial, arterial, venous, and abdominal pressure recordings that could be used for calibration of primary injury (energy deposition) models.

At present, the injury neurobiology data can be collected only from brain tissue necropsies at selected times from several anatomical locations in the brain. The next generation optical imaging with fluorescent labeling and microdialysis methods will enable collection of real time electrophysiology, biochemistry, and physiology injury data within the brain and the body. We believe that correlated experimental and computational animal models of TBI will be able to provide a link between the mechanical models and secondary injury/repair neurobiology models for which human only data will be too limited and too complex. Ultimately they may be able to establish scaling and extrapolation of injury and treatment protocols from animals to humans.

#### Validation on *in vitro* cell/tissue cultures

The use of animal models for studying brain injury may be restricted due to ethical and regulatory reasons. Furthermore, while the external mechanical loads in animal models can be well controlled, the internal cell/tissue biomechanics are difficult to monitor and quantify. The analysis of injury outcomes at the tissue/cell level requires animal sacrifices, tissue extraction, and may be affected by animal-to-animal variability. On the other hand, the *in vitro* cell cultures or brain tissue slices enable repeatable, controllable environments with direct access for optical, and electrophysiological measurements. The main requirement for *in vitro* neuro-injury models is that they should replicate the *in vivo* tissue biomechanics and post-injury sequelae. *In vitro* models of TBI have been used to study several aspects of neuronal pathobiology (Geddes and Cargill, 2001; Pfister et al., 2003; Lusardi et al., 2004; LaPlaca et al., 2005; Kumaria and Tolias, 2008; Chen et al., 2009; Lauret et al., 2009; Morrison et al., 2011), including metabolic and signaling events, neuroexcitation, hypoxia, and various targets for pharmacologic intervention (Kochanek, 2011). Conventional experimental *in vitro* models induce the neuronal injury by various methods such as direct deformation of the underlying elastomeric substrate, application of a rapid compression, fluid shear, mechanical transection, or direct micromechanical manipulators. Unfortunately, none of these completely represent the blast-induced biomechanical loads in a living brain, such as propagation of a steep fronted pressure wave which causes compression, tension, and shear waves. One way to achieve such conditions is to place the cell/tissue culture in a shock tube (Sawyer et al., 2011; Panzer et al., 2012b). One must be careful when performing *in vitro* shock tube tests that they are exposing the cell/tissue cultures to the loading that is witnessed inside the head and not in air. Compared to the *in vivo* brain, the *in vitro* neurotrauma models also have other limitations such as considerable variability in cellular morphology, lack of a vascular network, incomplete axonal myelination, low synaptic density, and use of much higher concentration of metabolites (Glc, O_2_) for culture maintenance. Nevertheless, at this time the *in vitro* models are probably the best platform to develop and validate mathematical models of secondary brain injury. To the best of our knowledge, with the exception of the primary biomechanics of the *in vitro* tissue, limited work has been documented on mathematical models of *in vitro* neurotrauma (LaPlaca et al., 2005; Morrison et al., 2006; Kaster et al., 2011; Prevost et al., 2011). Development of mathematical models of *in vitro* cell/tissue neurotrauma combining primary and secondary injury and repair models should be a priority for future research.

## Potential Applications and Future Opportunities

As in physics and engineering, mathematical modeling could play a major role in advancing our understanding of brain injury mechanisms, and help in neurodiagnostics, treatment, and protection. Development of a comprehensive mathematical model of brain injury, including blast TBI, is certainly feasible and necessary. Current state of the art models of blast waves and head/brain biomechanics provide an excellent foundation for the development of a primary brain injury model. More effort should focus on the development of mathematical models of secondary injury and repair mechanisms and on the link between the two. We also believe that a prototype of an integrated primary-secondary brain injury model can be developed within a few years, but it may require a concerted collaborative effort between biophysicists, neurobiologists, mathematicians, and experimentalists. Existing head anatomical/geometry models and validated CFD tools could be used to evaluate blast wave loading profiles on unprotected and helmeted human heads for various exposures. It would allow detailed analysis of loading pathways through anatomical regions including the eyes, ears, nose, and the role of protective armor (helmet, visors, hearing protection devices, and others). FEM models of primary biomechanics, validated on head phantoms and animal models, could provide a better understanding of how the blast load is transmitted to the brain, where the blast energy is deposited and how to design the protective armor to minimize the blast energy transmission to the brain. Predicted macroscopic tissue strains, strain rates and stresses may provide “initial conditions” for modeling microscopic tissue damage that could be correlated with injury thresholds from *in vitro* and animal experiments. The most challenging step is to link the models of the primary blast event with the resulting brain tissue damage including the secondary mechanobiology of injury and neuro-functional outcome. Such a modeling framework may become a foundation for a rational study of neuroprotection, diagnostics, and treatment. To achieve these goals future computational blast brain injury research should focus on the following aspects:

- High resolution computational models of a human and rat head/neck and articulated whole body models for blast wave and biomechanics simulations, specifically improved morphological resolution of brain structures such as CSF, sulci, gray/white matter, and vascular system
- Anatomic geometry and morphology of selected brain tissue structures such as cortical gray matter, dendritic/synaptic structures, and axonal network in corpus callosum, to support multiscale models of tissue micro-damage
- Benchmark quality, reproducible experimental models replicating blast injury mechanisms in animals, in *in vitro* cell/tissue cultures and human head physical phantoms to provide data for validation of blast biomechanics models
- Constitutive material models of high strain rate brain tissues for macro- and micro-biomechanics analysis of tissue damage and cavitation
- Improved numerical methods for modeling FSI events (e.g., brain-CSF interaction), cavitation, presence of vascular structures and enable long-time simulations
- Calibrated reduced order models for fast simulations of coupled primary injury biomechanics, cerebral hemodynamics, tissue perfusion, secondary injury, and repair mechanisms and structural and functional deficits
- Model based scaling of the injury and repair dynamics from *in vitro* to animals to humans
- Model-guided development of load- and tissue-specific brain injury criteria and thresholds and their effects on the neurological outcome
- Effective use of brain injury models to support diagnostics (e.g., biomarker kinetics), prescribed resting period and return to duty, development of drug targets, exploration of novel protection and treatment methods (e.g., hypothermia), and injury specific optimal pharmacology (pharmacokinetics, pharmacodynamics) and treatment (routes of administration, optimal time window, drug combinations, etc.)
- Support development of novel head (brain, ears, eyes) protective armor.

## Disclaimer

The views expressed in this paper are those of the authors and may not necessarily be endorsed by the U.S. Army or U.S. Department of Defense.

## Conflict of Interest Statement

The authors declare that the research was conducted in the absence of any commercial or financial relationships that could be construed as a potential conflict of interest.
